# Using 21^st^ century diagnostics to overcome barriers for lung function testing in primary care: it is time to consider oscillometry

**DOI:** 10.1038/s41533-025-00445-7

**Published:** 2025-08-11

**Authors:** Janwillem W. H. Kocks, Grietje H. Prins, Samuel Bardsley, Deesha Ghorpade, Sundeep Salvi

**Affiliations:** 1 General Practitioners Research Institute, Groningen, The Netherlands; 2 Observational and Pragmatic Research Institute, Singapore, Singapore; 3 University of Groningen, University Medical Center Groningen, GRIAC Research Institute, Groningen, The Netherlands; 4 Department of Pulmonology, University of Groningen, University Medical Center Groningen, Groningen, The Netherlands; 5 Academic Research, Pulmocare Research and Education (PURE) Foundation, Pune, India; 6 Symbiosis Medical College for Women & Symbiosis University Hospital and Research Centre,Symbiosis International (Deemed University), Pune, India

**Keywords:** Asthma, Chronic obstructive pulmonary disease, Diagnosis

## Abstract

Obstructive airways diseases, including asthma and COPD as the most commonly encountered respiratory diseases in primary care, are both diagnosed by lung function testing using spirometry as the gold standard. However, this test is not always available in primary care practices and if it is, it is a difficult test to perform, requiring extensive operator training and patient co-operation with multiple forced manoeuvres. These barriers can lead to underdiagnosis and misdiagnosis, which further contributes to increased suffering and mortality associated with asthma and COPD. Oscillometry uses oscillating pressure waves to measure airflow obstruction and provides an alternative diagnostic test which is quicker and simpler than spirometry, requiring little training and no forced manoeuvres. Moreover, it provides additional aspects of lung function measurement which are not obtained by spirometry, making it a valuable option in primary care for diagnosing asthma and COPD.

Screening and diagnosis of respiratory diseases benefits greatly from (physiological) lung function testing. Advances in the past decades have led to the availability of a multitude of lung function tests, all of which require correct execution and interpretation for optimal results.

Forced oscillation techniques, including impulse oscillometry, monofrequency forced oscillometry, and pseudorandom noise have been around for 60 years, but have only recently started gaining attention. Unlike spirometry, oscillometry is easier to perform for patients and healthcare professionals, requiring simple tidal breathing through the mouth for 15–30 s. Recent advancements have made oscillometry devices cheaper and more affordable.

Spirometry is the oldest lung function test and has emerged over the years as a diagnostic tool of choice for obstructive airways diseases. Despite the value of spirometry as an important diagnostic test, it is a difficult test to perform which requires a lot of patient cooperation and as a result, around half the patients are unable to perform a good quality test. It is time to reconsider behaviors that hold back progress and to take advantage of 21st century technology.

Broad implementation of oscillometry has been hindered by problems such as the lack of standardization of measurements, reference values and difficult interpretation of data, problems which have been largely resolved in the last decade, though there is still room for improvement.

With the publication of “technical standards for respiratory oscillometry”, steps have been taken to standardize oscillometry and collection of normative data^1^. Reference values have been developed by several groups across the globe, highlighting differences between populations, regions and devices^2^. The impact of race and ethnicity on oscillometry impedance measures remains difficult to characterize as most currently available prediction equations are derived from studies performed in Western Europe and China and to a lesser extent North America, Japan and Australia^3^. More recent arguments suggest that absolute cut-off values (similar to those used to define hypertension and hyperglycemia) are more applicable for oscillometry, providing an additional potential advantage over spirometry^4^. Several studies have identified cut-off values for bronchodilator response measured with oscillometry and have suggested the increased sensitivity of oscillometry can provide an earlier and greater measure of bronchodilator response compared to FEV_1_^5^. Data collection, especially from larger cohorts, will help to establish reference values (or cut-off values) required for interpretation in various populations. This process is ongoing as indicated by the doubling in number of publications on pulmonary oscillometry in the last 5 years.

Digital solutions, such as real-time feedback during oscillometry measurement, can function as quality control measures and support adherence to technical standards such as force of spontaneous breathing. Additionally, this could guide technicians through a (standardized) stepwise approach for normal oscillometry measurement, both improving the quality of measurements and comparability with other measurements. On a similar note, the historically complicated interpretation of oscillometry results can be facilitated by algorithms which have been developed. Such algorithms reduce required time and training for interpretation. The process can be further optimized by agreeing on an official, standardized way to interpret oscillometry results. Additional improvements will further lower barriers and improve ease of use, thereby increasing the possibility for widespread implementation.

Typically, most patients with asthma and/or COPD visit a primary care practice before, or instead of, a respiratory specialist^6^. Therefore, it is important that primary care has access to lung function measurements.

Although there have been efforts to increase the uptake of spirometry in primary care, the uptake has been disappointing^7^. While exact numbers are unknown, less than half of asthma and COPD patients undergo a spirometry measurement in primary care before initiation of treatment^8,9^. Possible reasons for the suboptimal implementation of spirometry are dependent on regional differences and range from a lack of understanding, training on proper use, time, availability or associated costs^10^.

To aid implementation of lung function testing in primary care, alternatives that overcome these barriers need to be found. Investigation into questionnaires and peak flow meters has shown that this combination is effective for screening for asthma and COPD, though additional lung function testing is required to provide a diagnosis, and questionnaires depend on symptom reporting by patients. While mini- and microspirometry tests are easier to perform by patients than regular spirometry, there are many patients who are unable to perform the test adequately. Additionally, patients and technicians require training to perform the measurement and measurements are not always accurate enough to yield a diagnosis^10,11^. Both peak flow measurements and spirometry alternatives are impacted by effort of the patient. In contrast, a clear advantage of oscillometry in the primary care setting is its simplicity: it requires only passive tidal breathing, minimal patient effort, and little operator training. While the overall diagnostic accuracy of oscillometry relative to spirometry is still under evaluation, especially for certain lung diseases, its ease of use makes it a powerful screening tool. By lowering the technical and logistical thresholds for testing, oscillometry has the potential to significantly increase the timely identification of asthma, COPD, and other respiratory conditions—particularly in settings where spirometry is unavailable or impractical.

Oscillometry is able to distinguish between healthy persons, obstructive airway diseases such as asthma and COPD and restrictive conditions such as pulmonary fibrosis, and also provides information about the degree of obstruction^5^. Therefore, it is well suited for diagnostic purposes. Even where spirometry is required (for example to enable prescription) oscillometry could have an important role in earlier diagnosis. For example, in China, oscillometry has been implemented as a screening tool for COPD. If oscillometry identifies airway obstruction, this can lead to a direct referral ahead of spirometry. This method rules out normal lung function before referral to spirometry, reducing patient load and burden. Furthermore, this approach has been enhanced by recently published predictive equations which integrate other factors including sex, age, height, and weight with oscillometry parameters to greatly increase the predictive accuracy to identify COPD in primary and community healthcare settings. The new predictive equation showed a high NPV (>90%) for spirometry-defined COPD, meaning that the risk of missing a patient with COPD after oscillometry and considering the factors above is low, and could be transformational for screening purposes^12^.

Despite advantages to oscillometry, there are some limitations to consider. Oscillometry measurements do not fully correspond with spirometry measurements as sensitivities of both techniques differ for respiratory diseases, meaning oscillometry might not detect abnormalities despite an abnormal FEV_1_ and the other way around^13,14^ which is more common with small airways disease especially. Another limitation is linked to the use of tidal breathing used for oscillometry measurement, as there are patient populations that only have obstructed breathing that is only present with forced breathing such as patients with emphysema. Suspected emphysema could be ruled out in those with normal oscillometry by performing a simple peak flow or FEV_1_ measurement. As with any diagnostic test (including spirometry), complex or ambiguous cases benefit from a combination of tests (such as computed tomography (CT) or body plethysmography) as well as considering the patient history to build a broad clinical picture and enhance diagnostic precision.

Although oscillometry is considered less effort-dependent and easier to perform, implying a shorter learning curve for operators and potentially quicker testing times, this is yet to be fully quantified in a side-by-side comparison with spirometry in a real-world setting. Further work is needed to assess the potential cost and time benefits for implementing oscillometry in routine clinical practice.

Oscillometry is a simple test to perform and provides more sensitive information than what spirometry does in patients with asthma and COPD. Small airways dysfunction found in early asthma and COPD is more sensitively picked up on oscillometry than spirometry. More recently, oscillometry has been shown to be useful for patients of interstitial lung disease and offers additional information on lung mechanics than what spirometry offers. In view of this, we believe that oscillometry would be a very useful diagnostic tool in primary care and would encourage physicians to use this widely.

## Data Availability

No datasets were generated or analysed during the current study.
